# Metabolic and perceived effort responses following acute multi-ingredient pre-workout supplementation prior to high-intensity functional training workouts

**DOI:** 10.3389/fnut.2026.1741204

**Published:** 2026-02-04

**Authors:** Gerald T. Mangine, W. Wil King, James W. Henley, Ashley Hines, Kristyn C. McGeehan, Jacob D. Fanno, Tiffany A. Esmat, John R. McLester, Jacob L. Grazer

**Affiliations:** Exercise Science and Sport Management, Kennesaw State University, Kennesaw, GA, United States

**Keywords:** cardiorespiratory, CrossFit, energy expenditure, HIFT, ultrasound

## Abstract

**Introduction:**

Multi-ingredient pre-workout supplements (MIPS) are formulated with ingredients thought to improve nutrient delivery, limit fatigue, and enhance focus. Since high-intensity functional training (HIFT) often challenges athletes to complete “*as many reps as possible*” (AMRAP) within a time limit, understanding the physiological and perceptual responses that underpin performance is important to success. However, little is known about the effects of any MIPS formulation on HIFT performance. Therefore, the purpose of this study was to assess the acute metabolic and perceptive responses following a 5- and 15-min HIFT AMRAP.

**Methods:**

Twelve men and ten women (29.3 ± 7.1 years, 171 ± 7 cm, 80.5 ± 15.6 kg) with at least 2 years of HIFT experience completed four weekly visits in a randomized, double-blind, crossover design. On each visit, pre-exercise heart rate, energy expenditure, subjective ratings of effort were measured, along with collecting ultrasound images of the rectus femoris (RF) and vastus lateralis (VL) to quantify muscle cross-sectional area (cm^2^, CSA) and echo intensity. Participants then consumed either the supplement (S) or placebo (P), rested 40 min, and then completed either a 5- or 15-min AMRAP circuit of rowing, barbell thrusters and box jumps. All pre-exercise measures were repeated after workout completion.

**Results:**

MIPS led to a greater increase in RF CSA in both workout durations (+1.22 cm^2^, *p* = 0.004; +1.43 cm^2^, *p* < 0.001) and VL CSA in the 15-min bout only (+4.63 cm^2^, *p* < 0 0.001). No other supplement-related differences were observed. Both workout durations elicited significant (*p* < 0.05) increases in muscle size, heart rate, blood lactate concentrations, oxygen consumption, caloric expenditure, and perceived effort, with proportionately greater in men than women.

**Conclusion:**

The multi-ingredient pre-workout supplement led to greater acute increases in quadriceps CSA, an indirect indicator of post-exercise muscle fluid shifts, without between-supplement differences in heart rate, energy expenditure, or metabolic byproducts. These findings help contextualize previously reported performance outcomes obtained using the same cohort and protocol.

## Introduction

Multi-ingredient pre-workout supplements (MIPS) are a popular category of ergogenic aids used by recreational and competitive athletes (1–3). Rather than relying on any single ingredient, their purpose is to enhance focus, exercise capacity, and performance through the combined effects of multiple compounds. Although ingredient combinations vary across formulations, common MIPS staples include caffeine, beta-alanine, creatine, and ingredients known to increase endogenous nitric oxide production (e.g., L-citrulline, beet root extract) (4). The individual effects of these ingredients are well documented (1, 2, 5–7). Some like caffeine, nitric oxide precursors, and osmotically active compounds are known to exert measurable physiological effects following a single acute dose (5–9), whereas others (e.g., creatine and β-alanine) are more commonly associated with chronic loading effects (6, 7). Still, evidence of acute benefits exists, and little is known about their synergistic effects when combined. Unfortunately, the inclusion of multiple ingredients inherently limits any ability to attribute observed outcomes to any single compound. Instead, studies on MIPS interpret outcomes based on the entire formulation. Such studies have shown various formulations to improve anaerobic power (10, 11), increase total volume completed in a resistance training session (10, 12–14) at a lower perception of effort (14), and extend time to fatigue during aerobic exercise (15). These findings support the use of MIPS to enhance single-modality exercise, particularly when sustained effort is required. However, training and sport do not always limit themselves to a single modality.

High-intensity functional training (HIFT; e.g., CrossFit) is a multimodal training strategy that variably combines exercises from weightlifting, gymnastics, calisthenics, plyometrics, and traditional aerobic training into a single workout-of-the-day (WOD) (16, 17). Although there are no rigid limitations for the design and programming of each WOD, most are written as circuits and aim to maximize density. That is, the most common instructions ask trainees to either complete ‘*as many repetitions as possible*’ (AMRAP) of a predefined circuit within a set time limit (i.e., the most amount of work within a given duration), or to complete predefined exercise prescription as fast as possible (i.e., a given amount of work in the least amount of time) (18–20). Higher repetition counts and faster times to completion depend on the trainee’s ability to complete repetitions quickly and efficiently, the number of autoregulated breaks they must take, the duration of those breaks, and the speed and efficiency of their transitions between exercises (19, 21, 22). A portion of the trainee’s strategic approach can be explained by their relative strength and skill (23), but their capacity to adhere to their strategy depends on energy availability over the duration of a WOD (19, 21–23). Herein lies the challenge. Studies have generally reported that most HIFT workouts elicit heart rate (>77% of maximal heart rate) and blood lactate concentrations (≥13.3 mmol/L) responses that fall within the defined ranges for high- or vigorous-intensity exercise (20). Sustained energy production is not expected from the energy pathways associated with vigorous effort (24, 25), at least, not for the length of typical WOD durations (i.e., 2–20+ minutes). Thus, enhancing energy availability through supplementation is a logical strategy for improving HIFT performance.

In a recent survey, more than 80% of HIFT trainees reported consuming at least one type of dietary supplement at least twice per week (3). One in four respondents stated they consumed supplements for the purpose of improving energy levels and performance, with approximately 20% using pre-workout formulations. Despite its prevalence in this population, there is very little evidence supporting the usage of MIPS to enhance HIFT performance (26). Over a decade ago, performance improvements were evaluated following 6 weeks consumption of a formulation containing pomegranate, beet root, tart cherry, green and black tea extracts (27). The authors noted improved performance in the second of two consecutive standardized WODs performed prior to and following the supplementation period. However, several of the study’s methodological choices make it difficult to explain why performance was improved in both workouts. For instance, the energy requirements between both workouts may not have been the same, and those of the first workout could have impacted the second workout’s energy requirements. Prescription was not modified between sexes, and their work was neither quantified nor verified to be equal. Furthermore, training experience and habits were not controlled, leaving open the possibility for skill to influence the results. A more recent study attempted to build upon many of these limitations in its investigation of another MIPS formulation and exercise duration on acute HIFT performance, pacing, and expressed kinetics (28). While the formulation did not affect repetitions completed in either version (5 or 15 min) of the same AMRAP, the authors observed that supplementation led to more work completed at a faster overall pace, and that these benefits mainly appeared during aspects of the workouts that required sustained, continuous effort. A shortcoming of that paper was that it did not pair performance results with any physiological responses to the MIPS formulation and exercises.

To better interpret these pacing- and work-based findings, it is necessary to consider physiological and perceptual responses that may plausibly underpin sustained work capacity during HIFT. Acute changes in muscle cross-sectional area (CSA) and echo intensity (EI), measured via ultrasound, are commonly used to characterize transient post-exercise alterations in muscle fluid content and quality (29–31), which may reflect changes in nutrient delivery, intracellular water, and early exercise-induced muscle stress. Such alterations could influence repeated force production and transition efficiency during continuous efforts. Post-exercise energy expenditure and respiratory measures provide complementary insight into early recovery kinetics and substrate utilization following high-density work bouts, particularly when direct physiological assessment during HIFT is not feasible (20, 32). Finally, perceptual responses such as effort and fatigue contextualize physiological strain within the athlete’s subjective experience and are known to influence pacing behavior, break frequency, and tolerance to sustained effort during HIFT-style workouts (19–22, 33, 34). Given established sex-based differences in muscle mass, substrate utilization, and physiological and perceptual responses to high-intensity functional training, sex represents an important contextual factor for interpreting acute responses to multi-ingredient pre-workout supplementation (20, 21, 35).

Therefore, the purpose of this companion paper was to expand on our previous report (28) by presenting additional data that examines the acute metabolic and perceptual responses to a commercially available MIPS formulation consumed prior to shorter (5-min) and longer (15-min) AMRAP-style HIFT workouts. Based on the inclusion of nitric oxide precursors and osmotically active ingredients, it was hypothesized that MIPS ingestion would primarily elicit greater acute increases in quadriceps CSA and lower EI compared to placebo, consistent with altered post-exercise muscle fluid dynamics and nutrient delivery. It was further hypothesized that improved nutrient delivery could shift energy provision toward a slightly greater aerobic contribution during and/or immediately following HIFT, which may manifest as lower blood lactate concentrations, a lower respiratory exchange ratio, and reduced perceived exertion and fatigue compared to placebo. Alternatively, it was anticipated that metabolic and perceptual responses could remain similar between supplement conditions despite performance benefits previously observed with the same cohort and protocol, reflecting improved efficiency rather than greater global physiological strain.

## Materials and methods

### Participants

Based on a previously observed effect of *f* (0.33) (14) and the following parameters (*p* < 0.05; *β* = 0.80) for four conditions completed in cross-over fashion, *a priori* analysis indicated that a minimum of 14 participants were needed for this investigation. Twenty-three men and women who possessed at least 2 years of HIFT experience were enrolled in this double blind, placebo-controlled crossover study. These were the same participants reported about in the study by Mangine et al. (36). All participants provided their written informed consent after a thorough explanation of study rationale, design, procedures and supplement ingredients. All participants were free from any injury or illness that could have impacted their performance and were not using any performance enhancing drugs or medications (as determined by PAR-Q+ and Health and Medical History Questionnaire). During the study, one male participant was removed due to scheduling issues that prevented him from completing his final visit within study timeline requirements. All other participants completed all study visits and reported no adverse events. This study was approved by the University Institutional Review Board (IRB-FY23-18).

### Study design

**Figure 1:**
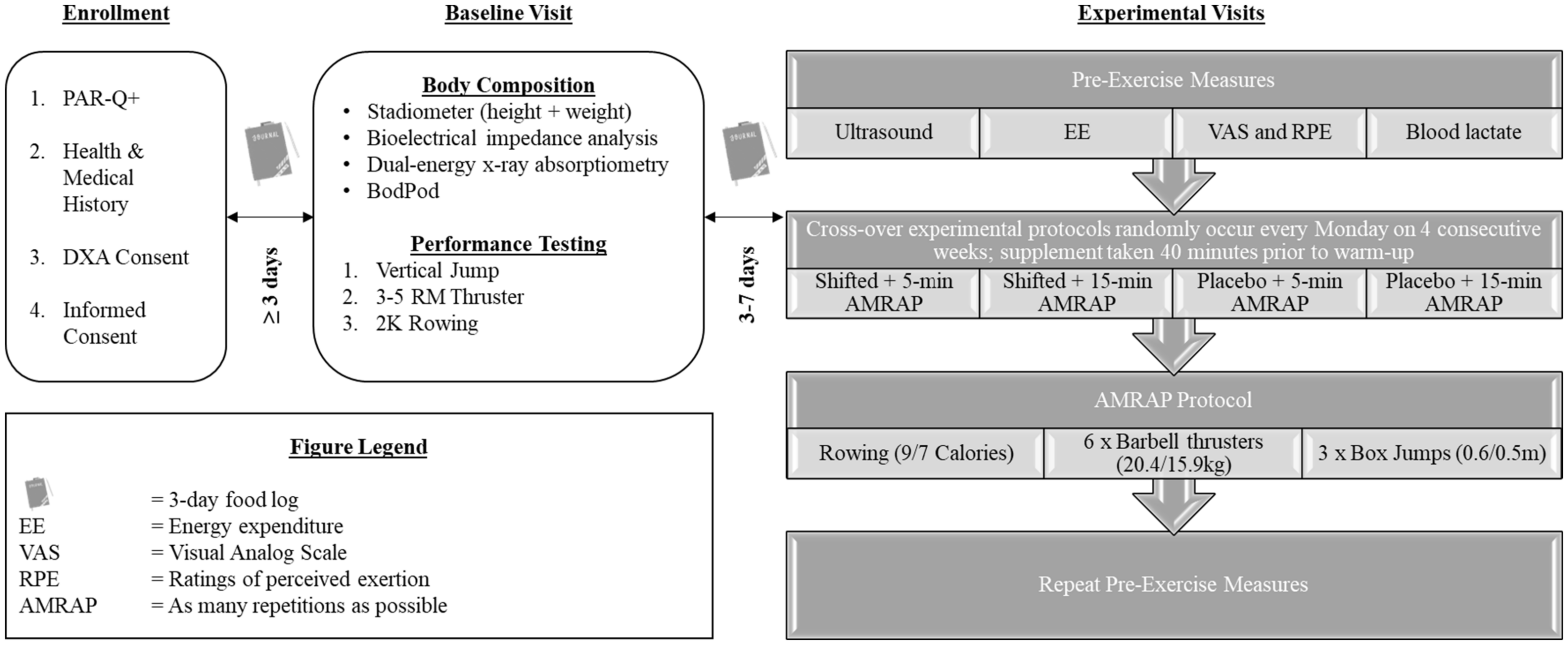
Study design.

The design of this study is illustrated in Figure 1. Participants were instructed to come to the Human Performance Laboratory (HPL) once per week, over the course of 5 weeks. All visits were scheduled on the same day each week during a time of day that was consistent with each participant’s usual workout time. Participants were instructed to avoid vigorous exercise within the 48-h leading up to each visit, and to arrive at the lab 2–3 h post-prandial, and to maintain their normal dietary behaviors for the duration of the study (verified by a 3-day food log). The first baseline visit had participants complete a four-compartment body composition assessment, followed by maximum vertical jump height, barbell thruster 1-repetition maximum strength, and 2,000-m rowing for time testing. A more in-depth explanation of the baseline visit protocol can be found elsewhere (28). Participants then completed the remaining four experimental visits in a randomized, counterbalanced order. Condition sequences were generated prior to data collection using a random sequence generator and were balanced across participants to minimize potential period effects. Each experimental visit had participants complete pre-exercise (PRE) measures of muscle size and quality, energy expenditure (EE), subjective perception of focus, energy, fatigue and perceived effort, and then blood lactate concentrations. Participants then consumed either the supplement (S) or placebo (P), and after resting 40 min and completing a standardized warm-up, were told to complete either a 5- or 15-min AMRAP WOD. The crossover study design ensured each participant completed all four conditions (i.e., 5-min AMRAP on S or P [5S or 5P]; 15-min AMRAP on S or P [15S or 15P]). All measurements collected at PRE were repeated post-exercise (POST).

### Ultrasound measurement

Ultrasound was used to collect non-invasive images of the *m. rectus femoris* (RF) and the *m. vastus lateralis* (VL). All images were collected from the left thigh to quantify muscle size and quality (via echo intensity), and these were measured prior to EE assessments at PRE and during EE assessments at POST. Due to likelihood of decreased post-exercise heart rate affecting the relevance of Doppler readings, changes in muscle size were used as a proxy for examining blood flow to muscle (i.e., muscle “pump”) (29, 31). Meanwhile, acute changes in echo intensity following exercise may reflect transient alterations in nutrient availability, intramuscular fluid shifts (cell swelling), and early indicators of muscle damage (30). Increased echo intensity (i.e., reduced quality) was assumed to reflect muscle edema, early damage, and the accumulation of metabolic by-products, whereas decreased echo intensity is thought to be consistent with enhanced perfusion or transient increases in intracellular water (cell swelling) and corresponds to improved nutrient delivery.

Participants initially laid quietly while a researcher identified the locations of the RF (50% of the distance between the proximal border of the patella and the anterior, inferior suprailiac crest) and VL (50% of the distance from the lateral condyle of the tibia to the most prominent point of the greater trochanter of the femur) using standard bony landmarks. This process took approximately 5–10 min and was essential for allowing fluid shifts to stabilize prior to image collection (37). All images were collected using a 12 MHz linear probe scanning head (General Electric LOGIQ S7 Expert, Wauwatosa, WI, USA) coated with water soluble transmission gel to optimize spatial resolution. Collection of each image began with the probe positioned on (and perpendicular to) the surface of the skin to provide acoustic contact without depressing the dermal layer. Subsequently, three consecutive images were collected in the extended field of view mode (Gain = 58 dB; Image Depth = 5–6 cm) using a cross-sectional sweep in the axial plane to capture panoramic images of each muscle. Each of these images included a horizontal line (approximately 1 cm), located below the image to assist in calibration during offline image analysis (38). RF images were collected while the participant laid supine with their legs extended but relaxed, whereas VL images required participants to lay on their side, with their legs stacked and position at a ~10° of knee flexion (32).

Images were transferred to a personal computer for analysis via Image J (National Institutes of Health, Bethesda, MD, USA, version 1.45 s). The polygon tracking tool in the ImageJ software was used to isolate as much lean muscle as possible without any surrounding bone or fascia from all panoramic images and quantify CSA (± 0.1 cm^2^) and EI (± 1 au). The intraclass correlation coefficients (ICC_3,K_), standard error of the measurement (SEM), and minimal difference (MD) of the researcher who directed the collection and analysis of all images for CSA (ICC_3,K_ = 0.88–0.99, SEM_3,K_ = 0.81–2.38 cm^2^) and EI (ICC_3,K_ = 0.74–0.95, SEM_3,K_ = 2.59–6.44 au) were previously collected in a resistance-trained population and reported elsewhere (39).

### Energy expenditure assessment

Upon arrival at the HPL, participants were fitted with a heart rate monitor (Team2, Polar, Lake Success, NY). Heart rate (HR; ±1 bpm) was tracked throughout the duration of each experimental session in both absolute and as a percentage of maximal HR (220 – age) with values after EE assessments (PRE), immediate post-exercise (IP), and 5-min post-exercise (5M) being used for all comparisons.

Participants were then asked to lay supine on a training table in a private room in the HPL. For EE assessments at PRE, participants were positioned underneath a ventilated canopy (i.e., clear plastic hood) and asked to remain motionless for approximately 10 min. The canopy was connected via tube to an air pump and metabolic measurement system (Parvo Medics TrueOne 2400, ParvoMedics Inc., Salt Lake City, UT) calibrated to a 16:1 ratio of percent oxygen to carbon dioxide. Relative volume of oxygen (RVO_2_, mL kg^−1^ min^−1^), relative volume of carbon dioxide (RVCO_2_, mL kg^−1^ min^−1^), metabolic equivalent of task (METs, RVO_2_ divided by 3.5 mL kg^−1^ min^−1^), respiratory quotient (RQ, RVCO_2_ divided by RVO_2_), and kilocalories were determined based on a 5-min interval of measured volume of oxygen consumption (VO_2_) with a coefficient of variation less than 10% (40). Participants repeated the EE assessment at POST, and this began within approximately 1 min of bout completion and lasted 10 min from that time point. However, the POST assessment replaced the ventilated canopy with a nose clip and a 2-way valve mouthpiece to avoid excessive accumulation of condensation within the canopy hood due to the participant’s sweat and elevated body heat post-exercise. A secondary metabolic measurement system, calibrated to a 16:4 ratio of percent oxygen to carbon dioxide and not connected to an air pump, was used during POST. The same metabolic variables were collected here during the range of time equating to 5–10 min post-exercise.

### Perceptive response scales

Participants completed subjective assessments of perceived effort and energy following the EE assessment at PRE and immediately at POST. A modified rating of perceived exertion (RPE) scale was used, with points ranging from 0 or “at rest” to 10 or “very, very hard,” and participants were instructed to circle the number that most closely reflected their current state. Additionally, participants were asked to provide a current subjective assessment of their feelings of focus, energy, and fatigue. To indicate their perception, participants were instructed to place a mark along a 15 cm line anchored with one extreme descriptor on the left (e.g., “No focus at all”) and another extreme descriptor on the right (e.g., “Maximum focus.”) (41). A ruler was later used to quantify the markings on the visual analog scale (± 0.01 cm).

### Blood lactate

Blood samples were collected at PRE following EE assessments, IP and 5 M on each experimental visit. One drop of blood (~50 μL) was collected each time using a single-use disposable lancet to pierce the side of the fingertip. A small aliquot of each blood sample (~0.7 μL) was transferred to a single-use lactate strip for lactate quantification (± 0.1 mmol L^−1^). The blood lactate analyzer (Lactate Plus Meter, Nova Biomedical, Waltham, MA) was operated by a trained researcher in accordance with manufacturer specifications.

### Supplementation

The S formulation was commercially available (SHIFTED Maximum Formula Pre Workout, SHIFTED LLC, Monteagle, TN, United States) and contained a variety of well researched ingredients at efficacious doses (see Table 1). The same researcher mixed one serving of this formulation with 10 fl. oz. of water prior to each participant’s arrival. The P formulation was prepared using commercially available MiO® Fit Liquid Water Enhancer (Kraft Heinz, Chicago, IL), a zero-calorie water flavoring product that contains water, natural flavors, citric acid, sucralose, potassium sorbate, and food coloring. It does not contain caffeine or anyother known ergogenic or stimulant ingredients. One serving of the “Berry Blast” was mixed with 10 fl. oz. of water to resemble S in taste and appearance. To help ensure that participants did not notice any small differences in color, taste or smell, S and P were consumed via an opaque shaker bottle while wearing a nose clip. To maintain a double-blind design and minimize potential influence on participants or other researchers, one researcher assigned and prepared the S and P before each visit. While these procedures were intended to reduce sensory cues, formal assessment of blinding success was not performed in the present study, and partial unblinding due to known sensory effects of ingredients such as caffeine and β-alanine cannot be excluded.

**Table 1:** Supplement ingredients.

Serving size: 1 scoop (30 g)		
Ingredients	Amount per serving	% DV
Calories	5	
Total carbohydrate	1 g	≤1%^*^
Niacin (as nicotinic acid)	15 mg	94%
Vitamin B6 (as pyridoxine HCl)	1 mg	59%
Vitamin B12 (as methylcobalamin)	100 mcg	4,167%
Iron	1 mg	6%
Magnesium (from red spinach leaf extract and dimagnesium malate)	9 mg	2%
Sodium (as pink himalayan sea salt)	40 mg	2%
Potassium (from red spinach leaf extract and potassium chloride)	248 mg	5%
L-citrulline	8 g	**
Creatine monohydrate	5 g	**
Taurine	3 g	**
Beta-alanine (as CarnoSyn®)	2.5 g	**
Betaine anhydrous	2.5 g	**
L-tyrosine	2 g	**
Red spinach leaf extract (as Oxystorm®)	1 g	**
Beet root extract	1 g	**
Alpha-GPC (alpha-glycerol phosphoryl choline 50%)	300 mg	**
Caffeine blend	300 mg	**
Caffeine anhydrous (250 mg)		
zümXR® delayed release caffeine (50 mg)		
L-theanine	150 mg	**
ElevATP® (ancient peat and apple fruit extract)	150 mg	**
Pink himalayan sea salt	**	**
*Rhodiola rosea* (root) extract	100 mg	**
Co-enzyme Q10	25 mg	**
AstraGin® [astragalus membranaceus (root) extract and panax notoginseng (root) extract]	25 mg	**
BioPerine® (black pepper fruit extract)	5 mg	**

*Percent Daily Values (DV) are based on a 2,000-calorie diet.

**Daily value not established.

Other ingredients: citric acid, natural flavor, calcium silicate, malic acid, silicon dioxide, sucralose, spirulina powder.

### Statistical analysis

All variables were separately assessed across all workout conditions (i.e., workout duration and supplemental condition) and time points using the linear mixed model procedure in SPSS (v.31, Chicago, IL, USA). All models used maximum likelihood estimation and an autoregressive-heterogenous repeated covariance to account for the dependent relationships existing between time points. Sex was added as a factor into the model due to known physiological differences (42) and expected differences in performance (18, 21, 22). Significant main effects were further examined by using the Bonferroni correction to compare pairwise differences between workout conditions. Significant interactions were further investigated by splitting the file by sex and repeating the linear mixed model procedure and by splitting the file by workout condition and performing independent *t* Tests to compare differences between men and women. All data are reported as mean ± standard deviation and statistical significance was set at *p* < 0.05.

## Results

### Muscle morphology

**Figure 2:**
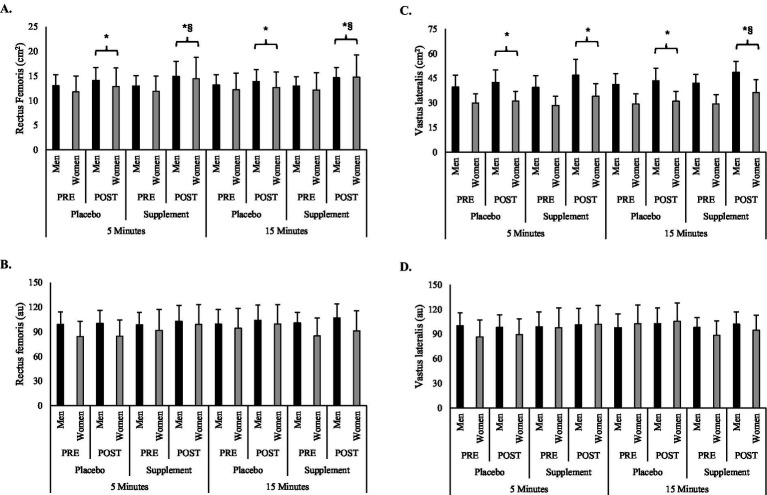
Changes in **(A)** rectus femoris cross-sectional area, **(B)** rectus femoris echo intensity, **(C)** vastus lateralis cross-sectional area, and **(D)** vastus lateralis echo intensity surrounding workout and supplemental conditions. ^*^Significantly (*p* < 0.05) different from PRE; ^§^Significantly (*p* < 0.05) different from placebo condition of same workout duration.

Main effects of condition were observed for RF and VL CSA (*F* = 22.5–31.7, *p* < 0.001). Muscle CSA (RF & VL) increased after all workout conditions, but greater increases were noted during supplement conditions. Greater increases in RF CSA were seen during both the 5-min (mean difference = +1.22 cm^2^, *p* = 0.004) and 15-min bouts (mean difference = +1.43 cm^2^, *p* < 0.001), whereas a greater VL CSA increase was only seen after the 15-min bout (mean difference = +4.63 cm^2^, *p* < 0 0.001). A main effect of sex (*F* = 20.4, *p* < 0.001) was seen with VL CSA being ~11.49 cm^2^ greater in men. No differences were noted about echo intensity. Changes in muscle morphology are illustrated in Figure 2.

### Heart rate and lactate responses

**Figure 3:**
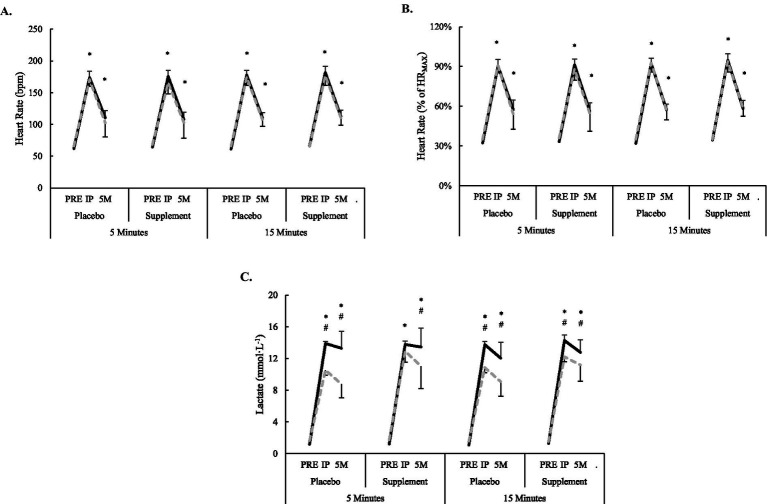
Changes in **(A)** absolute heart rate, **(B)** relative heart rate, and **(C)** blood lactate concentrations surrounding workout and supplemental conditions. Solid black bar (men); dashed grey bar (women). ^*^Significantly (*p* < 0.05) different from PRE for both men and women. ^#^Significantly (*p* < 0.05) different between men and women.

A significant interaction was observed for lactate (*F* = 8.4, *p* < 0.001), but this was primarily driven by sex differences. No differences were noted at PRE, and both men and women experienced significant elevations from PRE to IP (+10.1–12.7 mmol L^−1^, *p* < 0.001) and to 5M (+8.4–11.7 mmol L^−1^, *p* < 0.05). but concentrations were always significantly higher in men (mean difference = 1.6 mmol L^−1^, *p* < 0.001) except for at IP during 5S. Significant main effects for workout condition (*F* = 566–666, *p* < 0.001) showed HR (absolute and relative) were elevated from PRE at IP (mean differences = 110 bpm or 57.6% of HR_MAX_, *p* < 0.001) and 5M (mean differences = 43 bpm or 22.7% HR_MAX_, *p* < 0.001), but these were not different between supplement conditions or sexes. Changes in heart rate and blood lactate concentrations are illustrated in Figure 3.

### Energy expenditure

Significant interactions were seen for RVO_2_ (*F* = 3.3, *p* = 0.007), METs (*F* = 3.3, *p* = 0.007), and kilocalories (*F* = 5.7, *p* < 0.001). Increases from PRE to POST were noted for each but without any differences between supplement conditions. Post-exercise RVO_2_ (+1.44–1.52 mL kg^−1^ min^−1^, *p* < 0.05) and METs (+0.41–0.43, *p* < 0.05) were greater in men compared to women following the 15-min bouts but were the same after the 5-min bouts. Kilocalories were greater in men (+7.58–11.60, *p* < 0.05) following all workout conditions. Main effects of sex for RVCO_2_ (mean difference = +1.26 mL kg^−1^ min^−1^, *F* = 23.9, *p* = 0.004) and RQ (mean difference = 0.10, *F* = 34.9, *p* < 0.001) showed significantly higher values in men compared to women across all conditions and time points. Main effects of workout condition for RVCO_2_ (*F* = 23.9, *p* = 0.004) and RQ (*F* = 34.9, *p* < 0.001) showed no differences between supplement conditions, but instead, post-exercise elevations were seen for RVCO_2_ (mean difference = +3.87–4.30 mL kg^−1^ min^−1^, *p* < 0.001) and RQ (mean differences = +0.17–0.15, *p* < 0.05) following the 15-min bouts but only RQ was elevated after the 5-min bouts (mean difference = 0.27–0.29, *p* < 0.001). No other differences were observed. Changes in measures of energy expenditure are presented in Table 2.

**Table 2:** Changes in energy expenditure surrounding workout and supplemental conditions.

Variable	5 min				15 min			
	Placebo		Supplement		Placebo		Supplement	
	PRE	POST	PRE	POST	PRE	POST	PRE	POST
RVO_2_ (mL kg^−1^ min^−1^)								
Men	4.03 ± 0.60	7.29 ± 1.73*	4.03 ± 0.55	8.24 ± 2.04*	3.98 ± 0.41	7.77 ± 0.99*	4.00 ± 0.56	8.26 ± 0.79*
Women	3.89 ± 0.43	6.89 ± 0.96*	3.97 ± 0.53	7.24 ± 1.64*	4.00 ± 0.45	6.24 ± 1.28*#	4.04 ± 0.50	6.83 ± 1.75*#
Total	3.97 ± 0.53	7.12 ± 1.43	4.00 ± 0.53	7.80 ± 1.90	3.99 ± 0.41	7.10 ± 1.35	4.02 ± 0.52	7.61 ± 1.47
RVCO_2_ (mL kg^−1^ min^−1^)								
Men	3.68 ± 0.78	9.01 ± 2.59	3.57 ± 0.65	9.97 ± 2.74	3.50 ± 0.48	8.55 ± 1.93	3.63 ± 0.68	9.10 ± 1.47
Women^#^	3.32 ± 0.41	7.32 ± 1.89	3.26 ± 0.67	7.56 ± 2.01	3.39 ± 0.56	6.09 ± 1.61	3.42 ± 0.42	6.55 ± 2.12
Total	3.52 ± 0.66	8.27 ± 2.42	3.43 ± 0.66	8.93 ± 2.69	3.46 ± 0.51	7.48 ± 2.16^*^	3.53 ± 0.57	7.94 ± 2.18^*^
METs								
Men	1.15 ± 0.17	2.08 ± 0.50^*^	1.15 ± 0.16	2.35 ± 0.58^*^	1.14 ± 0.12	2.22 ± 0.28^*^	1.14 ± 0.16	2.36 ± 0.23^*^
Women	1.11 ± 0.12	1.97 ± 0.27^*^	1.13 ± 0.15	2.07 ± 0.47^*^	1.14 ± 0.13	1.78 ± 0.37^*#^	1.15 ± 0.14	1.95 ± 0.50^*#^
Total	1.13 ± 0.15	2.03 ± 0.41	1.14 ± 0.15	2.23 ± 0.54	1.14 ± 0.12	2.03 ± 0.38	1.15 ± 0.15	2.17 ± 0.42
Respiratory quotient								
Men	0.90 ± 0.10	1.23 ± 0.15	0.88 ± 0.08	1.21 ± 0.15	0.88 ± 0.06	1.09 ± 0.15	0.90 ± 0.06	1.10 ± 0.12
Women^#^	0.85 ± 0.05	1.06 ± 0.21	0.82 ± 0.09	1.06 ± 0.18	0.85 ± 0.09	0.97 ± 0.16	0.85 ± 0.05	0.95 ± 0.17
Total	0.88 ± 0.08	1.16 ± 0.19^*^	0.85 ± 0.09	1.15 ± 0.18^*^	0.87 ± 0.08	1.04 ± 0.16^*^	0.88 ± 0.06	1.03 ± 0.16^*^
Kilocalories								
Men	8.28 ± 0.89	26.55 ± 9.95^*^	8.31 ± 1.30	28.94 ± 8.56^*^	8.16 ± 0.84	26.92 ± 8.37^*^	8.30 ± 1.26	26.89 ± 5.07^*^
Women	6.26 ± 0.82^#^	18.96 ± 4.86^*#^	6.30 ± 0.78^#^	18.88 ± 5.71^*#^	6.40 ± 0.76^#^	17.21 ± 4.66^*#^	6.42 ± 0.66^#^	15.29 ± 5.66^*#^
Total	7.40 ± 1.32	23.25 ± 8.86	7.44 ± 1.48	24.57 ± 8.91	7.40 ± 1.19	22.70 ± 8.45	7.45 ± 1.39	21.62 ± 7.88

^*^Significantly (*p* < 0.05) different from PRE for both men and women; ^#^Significantly (*p* < 0.05) different from men.

### Perception of effort

Main effects of condition were noted for RPE (*F* = 116.4, *p* < 0.001) and fatigue (*F* = 16.3, *p* < 0.001). From PRE to POST, RPE (mean difference = +6.5–7.3 au) and fatigue increased (mean difference = +4.6–6.2 cm) across all workout conditions. A main effect of sex was also found for RPE (*F* = 23.9, *p* < 0.001) where men perceived the workouts as requiring more effort (mean difference = +0.77 au) than women. No other differences were seen. Changes in perception of effort measures are presented in Table 3.

**Table 3:** Changes in perception of effort surrounding workout and supplemental conditions.

Variable	5 min				15 min			
	Placebo		Supplement		Placebo		Supplement	
	PRE	POST	PRE	POST	PRE	POST	PRE	POST
RPE								
Men	0.62 ± 0.51	8.08 ± 1.85	0.69 ± 0.63	8.38 ± 1.76	0.82 ± 1.08	7.92 ± 1.83	1.00 ± 0.89	8.58 ± 1.16
Women^#^	0.60 ± 1.07	6.50 ± 1.84	0.89 ± 1.36	6.70 ± 1.25	0.80 ± 1.23	6.50 ± 2.46	0.40 ± 0.52	7.50 ± 1.51
Total	0.61 ± 0.78	7.39 ± 1.97^*^	0.77 ± 0.97	7.65 ± 1.75^*^	0.81 ± 1.12	7.27 ± 2.21^*^	0.71 ± 0.78	8.09 ± 1.41^*^
Focus								
Men	8.96 ± 2.34	9.95 ± 3.66	16.68 ± 25.46	11.13 ± 3.06	8.46 ± 2.30	10.03 ± 3.42	8.64 ± 2.68	9.78 ± 3.91
Women	8.22 ± 3.32	8.73 ± 2.56	8.16 ± 2.25	8.00 ± 2.71	9.07 ± 1.99	18.22 ± 31.32	8.68 ± 1.67	8.23 ± 3.55
Total	8.64 ± 2.77	9.42 ± 3.22	12.98 ± 19.35	9.77 ± 3.26	8.73 ± 2.14	13.59 ± 20.61	8.66 ± 2.20	9.07 ± 3.75
Energy								
Men	8.67 ± 2.93	7.60 ± 4.81	8.46 ± 3.35	7.74 ± 4.91	8.66 ± 2.81	7.18 ± 4.11	8.12 ± 3.10	6.37 ± 3.83
Women	7.93 ± 2.58	5.73 ± 2.36	7.44 ± 2.04	7.05 ± 3.39	9.18 ± 2.49	6.09 ± 3.06	9.22 ± 1.61	5.49 ± 3.87
Total	8.35 ± 2.75	6.79 ± 3.97	8.02 ± 2.84	7.44 ± 4.24	8.89 ± 2.63	6.70 ± 3.65	8.65 ± 2.51	5.97 ± 3.78
Fatigue								
Men	3.78 ± 3.06	10.05 ± 4.18	4.16 ± 2.68	10.89 ± 3.56	4.41 ± 2.84	10.98 ± 3.64	3.66 ± 3.02	11.70 ± 2.14
Women	5.39 ± 2.17	9.05 ± 2.91	4.79 ± 2.84	8.70 ± 2.45	5.63 ± 3.90	8.40 ± 3.79	5.75 ± 2.73	10.27 ± 2.72
Total	4.48 ± 2.77	9.62 ± 3.64^*^	4.43 ± 2.70	9.94 ± 3.25^*^	4.94 ± 3.31	9.86 ± 3.85^*^	4.65 ± 3.01	11.05 ± 2.47^*^

^*^Significantly (*p* < 0.05) different from PRE for both men and women; ^#^Significantly (*p* < 0.05) different from men.

## Discussion

The purpose of this study was to assess the acute metabolic and perceptive responses to a commercially available MIPS formulation after 5- and 15-min AMRAP versions of the same HIFT circuit. The data suggested that a single serving of MIPS acutely enhanced quadriceps CSA, but without effecting echo intensity or eliciting greater metabolic or perceived effort responses. To the best of our knowledge, only ten studies have been published on the ergogenic effects of any sports supplement on HIFT performance (28, 43) and only two have studied a MIPS formulation (27, 28). In the first study, Outlaw et al. (27) described the effects of 6 weeks of chronic supplementation on VO_2_max, Wingate power, and workout performance, whereas Mangine et al. (28) only reported on the acute effects of another formulation (the same formulation as this study) on workout performance. The present study builds on the latter by reporting the acute effects of the same formulation on related physiological responses. This study also adds to a limited body of knowledge about the effect of sex and AMRAP duration on acute physiological responses to HIFT (20, 35). Although men possessed larger muscle, utilized more energy, produced more metabolic byproducts (CO_2_ and lactate), and perceived their responses to both workout durations were proportional to responses shown by women. Meanwhile, regardless of sex, similar elevations in muscle size, heart rate, blood lactate concentrations, oxygen consumption, caloric expenditure, and perceived effort were seen following both workout durations. The only exception was the significant increase in CO_2_ expiration following the 15-min bout and not the 5-min bout. The findings of this study help to partially explain previously reported performances differences noted in connection with the present MIPS formulation (26).

The present MIPS formulation was selected because it includes ingredients known to enhance energy mobilization and delivery (1, 5–7, 44, 45) and mitigate fatigue associated with metabolic byproducts (e.g., hydrogen ions) (6, 7). Prior studies using this formulation reported improved high-intensity performance via greater total workloads during bench press (14) and HIFT-style AMRAPs (28). These findings were consistent with expected ergogenic effects, but the mechanisms remained uncertain. The greater acute increases in muscle CSA observed between S and P may partly explain these effects, as such changes are consistent with transient alterations in muscle fluid distribution that may influence nutrient delivery (29, 31). This formulation contains nitric oxide precursors (e.g., L-citrulline, beetroot, and red spinach extracts), which may promote vasodilation and facilitate oxygen and nutrient transport (8, 9, 46). Although not a direct measure, using ultrasound to assess muscle size and quality was the most practical option available in our laboratory for capturing these responses. Doppler assessments could have provided information about vessel diameter and blood flow, but ultrasound was necessarily delayed post-exercise. The collection of blood samples (for lactate analysis), RPE, and subjective perception measures were prioritized because these could be completed immediately. As expected, fatigued participants required ~60–90 s to walk to the recovery room (adjacent to workout room) after completing each workout, lie down and be connected to the metabolic cart. This was irrelevant to energy expenditure data because the initial few minutes were not going to be useful for objective comparisons (40). However, expected reductions in heart rate and perfusion within this time negated the utility of Doppler readings for assessing blood flow (47–49). Thus, acute increases in muscle size were viewed as reflecting residual blood flow rather than structural change. This interpretation is supported by the absence of differences in post-exercise heart rate and lactate concentrations. While reduced echo intensity would have strengthened this conclusion (30), variability in imaging depth and contrast settings across participants may have obscured such effects. Thus, the absence of direct perfusion measures limits mechanistic validation, and CSA findings should be interpreted cautiously.

The elevated heart rate and blood lactate values observed at IP and 5M on each experimental visit, especially in men, are consistent with the values summarized by McDougle et al. (20). A typical HIFT workout will elicit a post-exercise heart rate response equating to approximately 85–95% of maximal heart rate and blood lactate concentrations ranging from 13 to 19 mmol L^−1^, comparable elevations to those expected from maximal aerobic or Wingate testing. Their remaining elevated at 5M also aligns with established recovery kinetics following both shorter and longer duration HIFT workouts (50). These findings confirm that both durations imposed the characteristically high physiological stress of HIFT, which might explain the absence of differences between supplement conditions. With the cardiovascular and metabolic responses already approaching their physiological limits, their capacity for further modulation may have been minimal. The formulation included several ingredients known to impact cardiovascular and metabolic responses to exercise (e.g., caffeine, creatine, and beta-alanine) (5–7), but their effects are typically observed during submaximal exercise or after chronic supplementation. Caffeine can acutely elevate heart rate and modestly affect metabolic responses, but the responses might not be observable when exercise intensity already approaches maximal levels (5). Conversely, β-alanine, and creatine to a lesser extent, function as intracellular buffers and might be expected to attenuate blood lactate accumulation (6, 7). However, because workloads were not fixed between supplement conditions (i.e., participants were instructed to complete ‘*as many repetitions as possible*’ on each workout), greater metabolic strain during supplementation could have been offset by greater work output. Indeed, the same cohort completed more work during the rowing component and exhibited a trend toward faster thruster pacing, despite there being no differences in overall workout score between S and P (28). Rowing represented the longest and most continuous element and preceded the thruster segment, which may help explain why these task-specific performance effects were not reflected in global metabolic or perceptual differences. Acute lactate attenuation was not observed and is unlikely to meaningfully influence performance during near-maximal HIFT protocols. Any supplementation-related benefit is therefore more likely to depend on workout structure or chronic use rather than acute modulation of metabolic byproducts.

A similar argument can be made about why no differences were seen between supplement conditions. The post-exercise increases in energy expenditure variables (RVO_2_, METs, and kilocalories) most likely reflect excess post-exercise oxygen consumption rather than exercise intensity. These data were collected between 5 and 10 min after participants completed each workout, and the relatively low RVO₂ values (single-digit mL kg^−1^ min^−1^) are consistent with the tail end of recovery kinetics described in other HIFT studies (20). Men in this cohort possessed greater lean mass (28) and exhibited higher values in all energy expenditure variables. Men tend to exhibit more pronounced sympathetic activation and slower parasympathetic recovery following HIFT, and paired with their greater lean mass, they would proportionally require a higher oxygen cost during metabolic restoration (35, 50). Meanwhile, elevated RVCO₂ and RQ further indicate ongoing acid–base restoration (i.e., bicarbonate buffering of hydrogen) and a continued but temporary reliance on carbohydrate utilization (2, 25). The higher lactate, RVCO_2_, and RQ values observed in men are consistent with other HIFT studies (35, 48) and suggest a greater reliance on glycolytic energy pathways due to their having more lean mass. However, the relevance of these observations is limited to a very small post-exercise recovery window. There are inherent logistical difficulties associated with performing and transitioning between many common HIFT exercises while connected to portable or stationary metabolic measurement system. Many of the ingredients in the present formulation are either more commonly known to be effective after chronic loading or their effects on post-exercise recovery may not appear for several hours (5–9). Thus, it is possible that the near-maximal intensity of HIFT-style workouts limits the relevance of the present MIPS formulation for aiding in early phase recovery. As implied by Rios et al. (35), who also limited their attention to the acute recovery phase, metabolic recovery after HIFT may extend well beyond the short post-exercise window captured here. Elevated post-exercise metabolism may continue for many hours after completion (20, 25). Thus, the observed post-exercise energy expenditure response should be viewed as a contextual indicator of early recovery demand rather than a primary determinant of metabolic strain during the workout itself. Future studies should either monitor oxygen kinetics over a longer recovery period or explore alternative approaches for estimating energy expenditure during the workout itself.

Post-exercise perceived effort increased by 6.45–7.25 au and perceived fatigue by 4.67–6.18 cm, and these mirrored the responses observed in physiological markers and matched ranges (RPE = 6–8 au, Fatigue = 4–6 cm) reported in other HIFT studies (20). For RPE specifically, values were higher in men and this likely reflects greater physiological strain rather than motivational differences. They also exhibited higher post-exercise lactate concentrations and CO_2_ values, and elevated metabolite accumulation intensifies sensations of effort and discomfort (33, 34). Interestingly, perceived “energy” and “focus” did not change, which may reflect their vagueness and susceptibility to interpretation rather than their absence. Self-reported feelings are influenced by expectation, familiarity, and cognitive appraisal of effort (51–53), and while many ingredients in this formulation (e.g., caffeine and nootropics) might enhance mood under less demanding conditions, their effects likely diminish at near maximal intensities. The slightly higher RPE reported by men in the present study likely reflects their greater physiological demand, which is consistent with higher post-exercise heart rate, lactate, and CO_2_ output rather than differences in motivation or tolerance. The absence of supplement effects also agrees with the findings from Beyer et al. (14), who reported similar RPE values after a single dose of the same MIPS formulation during resistance exercise despite greater total training volume. Thus, both studies suggest that acute supplementation of the present MIPS formulation does not affect subjective effort at near-maximal intensity.

Although precision and repeatability of data collection within the context of a typical HIFT session heavily influenced methodological design, these choices must be considered when evaluating the data. Ultrasound was used to infer muscle perfusion because direct blood flow measurements were not feasible during or immediately after exercise. Direct assessment would have been too cumbersome and invasive during exercise, and fatigued participants would have varied in how quickly they could transfer themselves to the recovery room. Prioritizing this assessment could have also delayed other, more time-sensitive assessments such as blood sample collection for lactate analysis at IP and 5M, and POST energy expenditure values. The latter was an important consideration because energy expenditure could not be measured during exercise. HIFT organizes a variety of full-body movements into circuit-style workouts (16–20) that negate the utility of real-time assessment with any metabolic measurement system. Post-exercise assessment was the only reasonable choice, and there is an initial delay in when the system begins collecting valid data after beginning a measurement session (40). Furthermore, because the intention was to match the post-exercise time course used in other HIFT studies (20), energy expenditure was ultimately monitored within a short recovery window and when metabolic rates were already declining. This approach limits inferences about any supplement effects during exercise and does not comprehensively describe recovery. Subjective ratings of “energy” and “focus” were also difficult to interpret, as these terms may vary in meaning between individuals (51–53). However, these were chosen to match the previous study using the same MIPS formulation (14). Future investigations would benefit from using more narrowly defined and validated constructs, such as task-specific mental fatigue, attentional control, or cognitive workload, to better characterize perceptual responses during HIFT. Finally, although participant characteristics were well-documented here and elsewhere (28, 36), there is no formal system for classifying this population. It is possible that within-sample variability could have masked the true effects of the supplement. Future research should continue to endeavor in providing detailed participant descriptions for the purposes of developing a classification system and help ensure fair comparisons are being made in any HIFT study. Regarding the current MIPS formulation, questions remain about the potential impact of chronic supplementation and what occurs over the course of a longer recovery period.

This study sought to examine the acute metabolic and perceptive responses to a multi-ingredient pre-workout supplement during HIFT-style workouts. Both workout durations produced expected cardiovascular and metabolic responses, as well as differences between men and women. The data suggests that the supplement may acutely enhance muscle perfusion, as indicated by increased quadriceps size, without altering heart rate, lactate, energy expenditure, or perception responses. These findings help explain the performance improvements previously reported about this cohort (28). However, practical implications for acute, single-dose use should be interpreted cautiously and within the context of workout structure and training demands. The present MIPS formulation appears to enhance the capacity to do more work during continuous-effort tasks, potentially through transient alterations in muscle fluid dynamics that may support nutrient delivery to active muscle. Although the supplement was equally beneficial for both shorter (5 min) and longer (15 min) AMRAP circuits, this effect may not generalize across all possible HIFT programming designs.

## Data Availability

The raw data supporting the conclusions of this article will be made available by the authors, without undue reservation.

## Author’s note

“All authors confirm that the following manuscript is a transparent and honest account of the reported research. This research is related to a previous study by the same authors titled “Acute effect of a multi-ingredient pre-workout supplement on pacing and kinetic expression during shorter and longer bouts of high intensity functional training”. The previous study was performed on the same participants and reported on performance outcomes and exercise kinetics in response to the multi-ingredient pre-workout supplement. The current submission focuses on the acute physiological responses to the same formulation within the context of the same study design.”
